# Gitksan medicinal plants-cultural choice and efficacy

**DOI:** 10.1186/1746-4269-2-29

**Published:** 2006-06-21

**Authors:** Leslie Main Johnson

**Affiliations:** 1 Centre for Work and Community Studies and Centre for Integrated Studies, Athabasca University, 1 University Drive, Athabasca, Alberta, Canada; 2 Anthropology Department, University of Alberta, Edmonton, Alberta, Canada

## Abstract

**Background:**

The use of plants for healing by any cultural group is integrally related to local concepts of the nature of disease, the nature of plants, and the world view of the culture. The physical and chemical properties of the plants themselves also bear on their selection by people for medicines, as does the array of plants available for people to choose from. I examine use of medicinal plants from a "biobehavioral" perspective to illuminate cultural selection of plants used for medicine by the Gitksan of northwestern British Columbia, Canada.

**Methods:**

Consultant consensus, "intercultural consensus", independent use of the same plants by other cultural groups, and phytochemistry and bioassay results from the literature, were employed in analysis of probable empirical efficacy of plant uses.

**Results:**

70% of 37 Gitksan medicinal plants were used similarly by other cultures where direct diffusion is not known to have occurred; eleven plants, including the eight most frequently mentioned medicinal plants, also show active phytochemicals or bioassays indicating probable physiologically based therapeutic effects.

**Conclusion:**

Analysis of intercultural consensus revealed that the majority of cultures in the British Columbia region within the plant ranges use the same plants, or closely related species, in similar ways. The rigor of this analysis is effected by the lack of consistent data on all taxa of interest for all cultures within the region.

## Background

The use of plants for healing by any cultural group is integrally related to local concepts of the nature of disease, the nature of plants, and the world view of the culture. The physical and chemical properties of the plants themselves also bear on their selection by people for medicines, as does the array of plants available for people to choose from. I have used a biobehavioral approach to analysis of Gitksan medicinal plant use, examining both what is known of the plants and their pharmacological properties, and Gitksan healing perspectives and practices, to attempt to understand why people are selecting certain plants as medicines.

**Figure 1:**
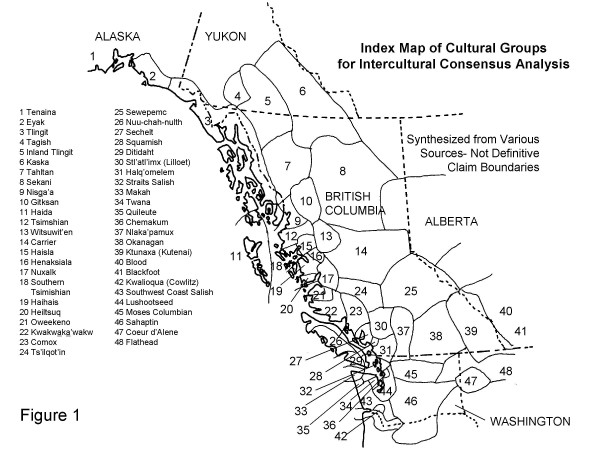
Index Map of Cultural Groups for Intercultural Consensus Analysis.

The Gitksan (Fig. 1) live in northwestern British Columbia in a heavily forested region. Many of their medicines are made from barks of trees or shrubs. The most frequent type of Gitksan medicine preparation is the mixed decoction of barks called **haldowkumgan**, which means "wood medicine" or "bush medicine". Needles or leaves such as pine (*Pinus contorta *Dougl.) or spruce (*Picea *× *lutzii*) tips are also used, pounded with pitch or grease for salves and in mixed decoctions drunk as medicinal teas. Whole herb plants may also be used for decoctions or poultices. Roots or rhizomes such as cow parsnip root (*Heracleum lanatum *Michx.) may be applied as poultices to sore joints or fractures, and inner barks may be used as wound dressings. Plants are also burned as smudges and used in the sweat hut **anguxw'uutx**; Gitksan healing practice includes steam baths with herbal washes or herbs burned on the hot rocks. Plants can be used as charms for protection or luck. Internal, external or spiritual cleansing may be a goal of plant use. Purgative and emetic preparations are often referred to in English as "cleansers".

Gitksan ethnomedical concepts spring from an understanding of humans as a part of all nature, and in reciprocal relations with all other entities, including sources of foods and potentially medicinal plants. Gitksan healing is holistic. Other key concepts are that of balance -avoidance of excess, and respect, for self and for others, which are seen as important in living a good life and in maintaining health. As an aspect of respect, attitude is important in collection and use of plants, which are seen as entities with their own intrinsic power to help and heal, or to withhold help.

Gitksan medicines are often slow acting, and may be integrated into life over prolonged periods. Use of tonics to promote health and prevent sickness is common. For the Gitksan, the underlying cause of much illness is seen to be spiritual, or a result of imbalance. The concepts of purification and cleansing are significant in both healing and gaining power. For a person who is ill, cleansing allows a return to balance and reinstatement of health. For a person who seeks supernatural power, cleansing removes dirt and impurity and may enable the seeker to gain power; "dirt" is specifically cited as a barrier to power in some teachings.

There are material and non-material aspects to all things in the Gitksan view. As a corollary to this, smudging or burning to release non-material essences is a culturally important mode of using some medicinal plants, especially those which are seen to have spiritual efficacy.

Formerly the Gitksan used elaborate shamanic curing ceremonies in conjunction with herbal medications, fasting, cleansing, prayer, and means of ensuring luck and personal power. There were shamanic healing specialists called **halaaydim swanaasxw**, who relied on ritual and non-material means to diagnose and cure illness in public ceremonials [1,2]. There is still use of some ritual or spiritual types of healing, especially within family contexts, as well as adoption of newer spiritual healing methods like Christian prayer meetings and Revivals.

## Methods

The present analysis of Gitksan medicinal plant use is derived from my ongoing fieldwork with the Gitksan from the period 1985–1998. Information on plant uses and traditional healing was gathered from 60 people in more or less formal contexts. Thirty-one consultants were women, and twenty-nine were men. The majority (49) were over 50 at the time of interviewing. Ethnomedical data were gathered in the course of interviews on the topic of plant uses, and in several interviews specifically on the topics of shamanic practice and traditional healing. Perspectives on health and healing were also shared in the course of informal conversation. The project was initially undertaken while working for the Gitksan-Wet'suwet'en Education Society, and everyone who participated was informed of the nature of the project. Participation was strictly voluntary, and the consultant's wishes were followed in terms of what information was recorded and whether it could be shared. Subsequently, I obtained ethics clearance from the University of Alberta while a graduate student there for the continuation of the project. Since that time I have continued to consult with individual Elders and teachers, and with the Gitxsan Tribal Council [Now Hereditary Chief's Office]. Original tapes and other materials are on deposit in Hazelton, BC in the Hereditary Chief's Office library. Plant specimens have been deposited in the Herbarium of the Botany Division of the Royal British Columbia Museum, and in the Vascular Plant and Cryptogamic Herbaria of the University of Alberta (see list of specimens in Table 5). The identifications have not been confirmed by taxonomic specialists. Earlier works which report on aspects of Gitksan ethnobotany and medicinal plant use include [3,1,4], and the recently published work of Harlan Smith [5]. A more complete discussion of methodology will be found in [2].

In my analysis of Gitksan medicinal plant use, I have combined the biobehavioral methods of Carol Browner, Bernard Ortiz de Montellano and Arthur Rubel [6]with the informant consensus approach pioneered by Robert Trotter and Michael Logan [7] in a study of Hispanic folk medicine in Texas. I also use what I call "intercultural consensus" as an additional approach to confirmation of plant uses, which is helpful in areas where extensive trade and intercultural contact make it impossible to assume that each culture may have independently chosen healing plants and techniques for their use.

Given the difficulty of ensuring that use of plants could not have been learned by contact with neighbouring groups who use the plant, David Young and his colleagues at the University of Alberta have tried to look at use of the same plants in widely separate geographic localities (Alberta and China) (Young *et al*. unpublished manuscript.). Such an approach limits analysis to plant species of very wide distributions, but many plants of medicinal interest are more restricted in range. Devil's club (*Oplopanax horridus *(Sm.) T.&G. ex Miq.) exemplifies the problems of restricted range in confirmation of efficacy by independent use by other cultural groups, as its range is restricted to northwestern North America, a region which has seen extensive cultural interchange over a long period of time. I have attempted instead to ascertain relative intercultural consensus in the use of plants for medicine as an analogue to intracultural consultant consensus for confirmation of the likely empirical efficacy of plant remedies (cf. [7]).

Intercultural consensus is agreement in which plants are valued for medicinal use, and similarity of reported uses between cultures which are in contact through such activities as trade, feasting and intermarriage. Heinrich et al. [8] approach intercultural comparison in a more geographically restricted and structured format in their work on medicinal plant choice in four Mexican indigenous groups, but their approach requires a series of parallel studies with the same methodology and classification of symptomology to be applicable. My method of determining intercultural consensus has been to map records of similar use of a given plant for medicine by all groups for which information is available within the range of the plant within my map area of northwestern North America from the Columbia River to the mainland of Alaska (Figure 1). [9], personal field notes, informal communication with colleagues, and references in Johnson [2] were used in this analysis. This approach is intended to supplement truly independent records of use by groups not in any direct contact, which can be obtained for plants with wide ranges in North America or globally.

The methodology of Browner and her associates involves examining the degree to which empirical criteria confirm the probable efficacy of the ethnomedical use or practice based on a biomedically grounded understanding of human physiology in the context of the ethnomedical concepts of the cultural group. While an approach grounded in physiology and phytochemistry is not the only way to assess healing efficacy, it is a useful perspective and helps cross cultural comparison. Their lowest level of empirical confirmation is Level 1, which requires "reports of parallel usage in populations among whom diffusion is unlikely" suggesting that "chemical activity exists" [6]:686.

For a medicinal plant use to be confirmed at Level 2 in Browner *et al*.'s methodology requires chemical analyses which verify presence of active phytochemicals which could produce a therapeutic physiological effect OR in vitro bioassays suggesting a therapeutic effect. Level 3 requires in addition a plausible mode of action which would likely produce a desirable therapeutic effect in a living patient. To reach their Level 4, clinical studies supporting the effective use of the phytochemicals or common use of those compounds in biomedicine are required.

## Results

Most of the medicinal plants used by the Gitksan are also used by at least one other culture in a similar manner, where direct diffusion is not known to have occurred, and are confirmed at Level 1 (Table 1). (In one instance a plant use that was not confirmed by another cultural group may be confirmed by the presence of an antiinflammatory compound that likely would be helpful in treatment of fractures, even though no other groups used it in a similar way). This tabulation includes use by both distant groups where true independent discovery can be assumed, and groups from the general northwestern region of North America, where chains of contact and cultural and cosmological similarities may have influenced choice and use of medicinal plants. The use of eleven of these plants is also confirmed at Level 2 or higher by the presence of active phytochemicals or bioassays which show potentially therapeutic activities (Table 2).

**Table 1:** Empirical Confirmation of Gitksan Medicinal Plant Uses – Level I

			Confirmed
Latin name(s)	Used by Other Groups	Similar Uses	Level 1
*Abies lasiocarpa *(Hook.) Nutt.	12	yes	**√**
*Achillea millefolium *L.	53	yes	**√**
*Alnus crispa *(Drylander ex Ait.) Pursh ssp. *sinuata *(Regel) Hultén	5	yes	**√**
*A. viridis *(Chaix) DC.	2	yes	
*Alnus incana*(L.) Moench	14	yes	**√**
*A. rubra *Bong.	10	yes	**√**
*Anemone ?multifida *Poir.	4	yes	**√**
*Angelica genuflexa *Nutt.	1	**NO**	**NO**
*Aralia nudicaulis *L. &/or	6	yes	**√**
*Actaea rubra *(Ait.) Willd.	15	yes	**√**
*Arnica cordifolia *Hook.	3	NO*	**NO**
*Athyrium filix femina*(L.) Roth &	9		**√**
*Drypteris filix-mas *(L.) Schott	3		**NO**
*D. expansa *(K.B.Presl) Fraser-Jenkins & Jermy	4		**√**
*Calla palustris *L.	2	**NO**	**NO**
*Castilleja miniata *Dougl.	**NO**	NO*	**NO**
*Cornus stolonifera *Michx.	23	**NO**	**NO**
*Equisetum arvense *L. &	19	yes	**√**
*E. hyemale *L.,		yes	**√**
*E. variegatum *Schleich,		yes	**NO**
*E. pratense *Ehrh.		yes	**√**
*Geum macrophyllum *Willd.	11	yes	**√**
*Heracleum lanatum *Michx.	30	yes	**√**
*Inonotus obliquus *(Pers:Fr.) Pilat &*Fomes ignarius*	4	yes	**√**
*Juniperus communis *L. &	34	yes	**√**
*J. scopulorum *Sarg.	17	yes	**√**
*Ledum groenlandicum *Oeder	20	yes	**√**
*Lobaria pulmonaria *&*L.oregana*	3	**NO**	**NO**
*Lonicera involucrata *(Rich.) Banks	13	yes	**√**
*Lupinus arcticus *Wats.	**NO**	**NO**	**NO**
*Malus fusca *(Raf.) Schneid.	11	some	**√**
*Nuphar polysepalum *Engelm.	10	yes	**√**
*Oplopanax horridus *(S.) T.&G. ex Miq.	36	yes	**√**
*P*.x *lutzii*, hybrid(Roche) spruce-	36**	yes	**√**
*P. engelmanii *Parry x		yes	**√**
*P. sitchensis *(Bong.) Carr x		yes	**√**
*P. glauca *(Moench) Voss		yes	**√**
*P. mariana *(Mill.) Britt., Sterns & Pogg.	6	yes	**√**
*Pinus contorta *Dougl.	24	yes	**√**
*Populus tremuloides *Michx.	19	some	**√?**
*Prunus pensylvanica *L.	6	yes	**√**
*Sambucus racemosa *L.	22	yes	**√**
*Shepherdia canadensis *(L.) Nutt.	16	yes	**√**
*Smilacina racemosa *(L.) Desf.	20	yes	**√**
*Sorbus sitchensis *Roemer and *S. scopulina *Greene	9	yes	**√**
*Thalictrum occidentale *Gray	**NO**	NO*	**NO**
*Tsuga heterophylla *(Raf.) Sarg.	12	**NO#**	**NO**
*Veratrum viride *Ait.	27	yes	**√**
*Viburnum edule *(Michx.) Raf.	5	some	**√**

√ = confirmed at Level 1

*related species similar use

#confirming record not independent

**Table 2:** Empirical Confirmation of Gitksan Medicinal Plant Uses Higher Than Level I

*Species*/illnesses	Level	Number of Groups Using	Number of Consultants
*Abies lasiocarpa*		12	9
respiratory illness	3		
tonic	2		
*Alnus incana/A. rubra*		14/10*	2
skin ailments	3		2
*Equisetum arvense/E.hiemale*		5/12	1,1
*E. arvense *diuretic, kidney	3 or 4		1
*/E. hiemale *diuretic, kidney	2 or 3		1
*Geum macrophyllum*		11	1
sores (if fungal)	3		
*Juniperus communis*		27	8
respiratory illness	3		
tonic	3		
*Nuphar polysepalum*		10	17
tuberculosis	2 or 3		
*Oplopanax horridus*		36	29
respiratory illnesses	2 or 3		4
tuberculosis	2 or 3		4
wound dressing	2		3
skin wash	?2		2
diabetes	?4		2
*Picea glauca*		36***	9**
tonic	2		
respiratory illness	2		
wounds, sores, burns	2		
*Pinus contorta*		24	9**
colds and respiratory illness	2 or 3		
wounds, sores, burns	3		
"sickness"	2		
*Sambucus racemosa*		22	5
emetic	2 or ?4		
*Veratrum viride*		27	14
skin	2 or 3		2
boils & swellings	2		****
aches, pains (topical)	2		****

* the numbers refer to *Alnus incana*- 14 reports and *Alnus rubra*- 10 reports

** includes both pine and spruce

*** The spruce used by the Gitksan is a hybrid swarm derived from *P. sitchensis*, *P. engelmannii *and *P. glauca*.These three species together are used by 18 groups.

**** These uses are reported in Harlan Smith's data from the 1920's [5] and were not provided by contemporary consultants.

Consultant consensus analysis of interview reports from 29 Gitksan consultants revealed eight medicinal plants which were mentioned by five or more individuals (Table 3). I have grouped use of spruce and use of pine in this tabulation, because many of their uses are interchangeable. These data are derived from unstructured interview notes, and therefore are likely an underestimate of the actual degree of consensus among knowledgeable Gitksan. The 29 consultants, including both men and women, came from all of the Gitksan villages and therefore represent a broad sample of current Gitksan medicinal plant knowledge.

**Table 3:** Consultant Consensus, Most Frequently Mentioned Gitksan Medicinal Plants

Plant Species/Uses	Number of Consultants
*Oplopanax horridus*	**29**
tonic	7
respiratory illness or colds	4
TB	4
arthritis	4
blood/heart	4
purification, enhance luck	4
stomach, stomach ulcer	3
wounds, skin ulcer	3
cancer	3
headache	2
flu, stomach flu	2
cleanser, purgative	1
cure-all	1
smudge	1
*Nuphar polysepalum*	**17**
TB	6
fractures (poultice)	4
arthritis (poultice)	3
birth control (male)	2*
"sickness"	2
sores, wounds (poultice)	3
appetite stimulant (especially for TB)	1
aches & pains	1
liver	1
intestines	1
tonic	1
*Veratrum viride*	**13**
purification/smudge	9
protection (amulet)	5
hunting luck	5
bath (add to bathwater)	3
skin & hair	2
snuff for sinuses	2
"germ killer" (removes contamination)	2
nightmare remedy	1
sleepwalk treatment	1
*Abies lasiocarpa*	**9**
TB (mixtures or pitch)	4
tonic (mixtures)	3
purgative (pitch)	2
cuts or boils (pitch)	2
internal, intestine, liver	2
lung disease	1
arthritis (as cleanser)	1
*Picea *× *lutzii *and *Pinus contorta*	**9**
burns, boils, infected cuts, salve	9
tonic, 'wood medicine' (mixtures)	4**
TB	1
*Juniperus communis*	**8**
tonic (mixture)	2
'wood medicine' mixture	5
smudge (can be mixt.)	2
*Sambucus racemosa*	**5**
emetic (for treatment of inability to eat, flu)	5
smudge, mixt. for spiritual medicine	1

Plants included on this table were mentioned by five or more consultants in analysed interviews and fieldnotes recorded from 1985–1997

* Harlan Smith (n.d.) also reported this use

** 1 tonic, 3 'wood medicine'

**Figure 2:**
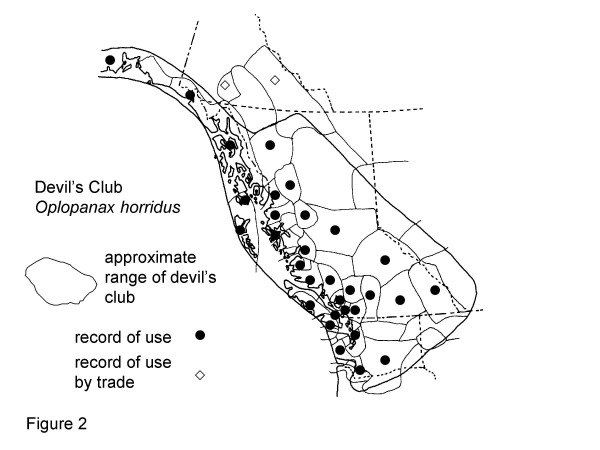
Map of Medicinal Use of Devil's Club, *Oplopanax horridus*.

**Figure 3:**
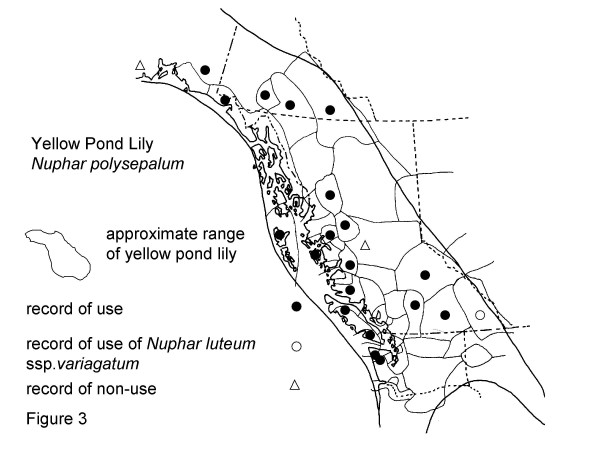
Map of Medicinal Use of Yellow Pond Lily, *Nuphar polysepalum*.

**Figure 4:**
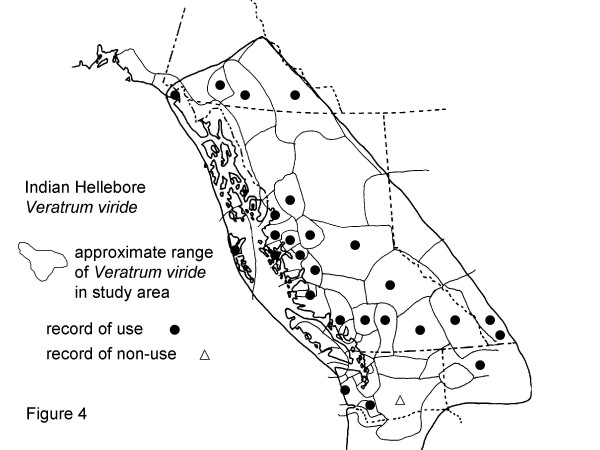
Map of Medicinal Use of Indian Hellebore, *Veratrum viride*.

**Figure 5:**
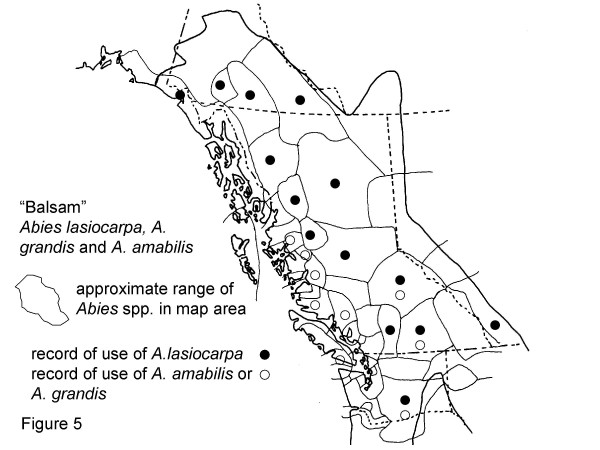
Map of Medicinal Use of "Balsam", Abies lasiocarpa, *A. grandis *and *A. amabilis*.

**Figure 6:**
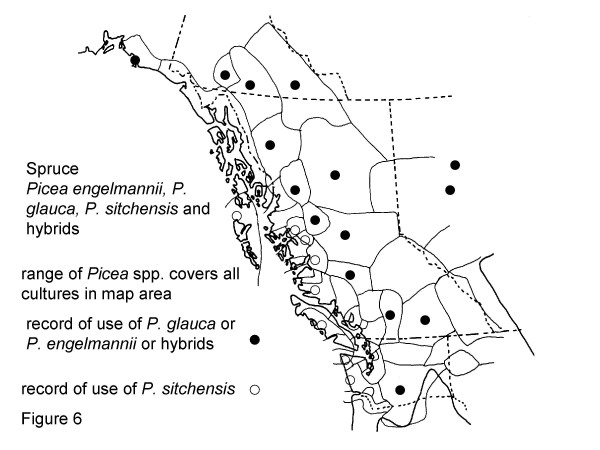
Map of Medicinal Use of Spruce, Picea engelmannii, *P. glauca, P. sitchensis *and *hybrids*.

**Figure 7:**
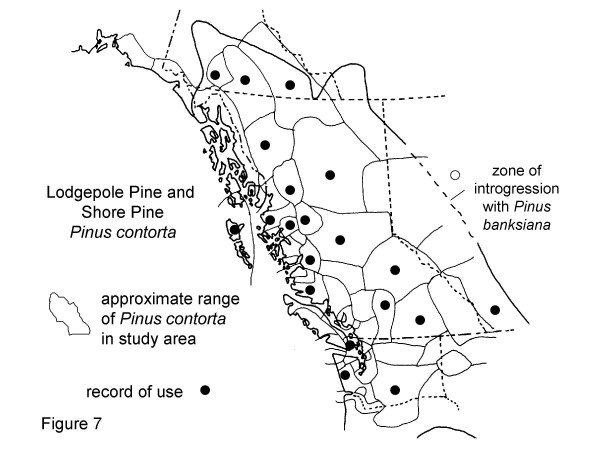
Map of Medicinal Use of Lodgepole Pine and Shore Pine, *Pinus contorta*.

**Figure 8:**
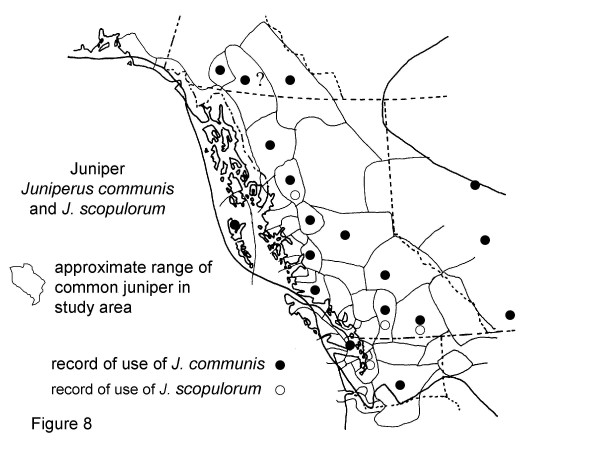
Map of Medicinal Use of Juniper Juniperus communis and J. scopulorum.

**Figure 9:**
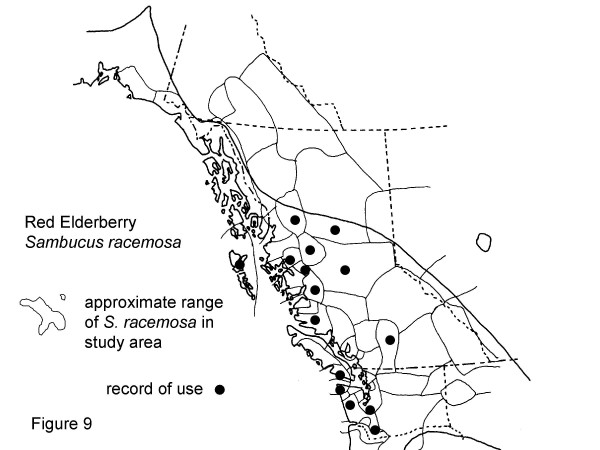
Map of Medicinal Use of Red Elderberry, *Sambucus racemosa*.

The eight plants most frequently mentioned by Gitksan consultants are, interestingly, all confirmed at Level 2 or greater for at least some of their traditional uses (Table 2). Though these data are suggestive, the phytochemical data are rarely sufficient to indicate amounts likely to be taken in actual use, nor are many of the plants adequately analysed to know what may be in them. Similarly, many phytochemicals known to occur in plants have never been investigated for their pharmacological activities. Nonetheless, confirmation at Level 2 or higher is indicative possible modes of physiologic action of traditional plant medicines. Intercultural consensus also supports likely efficacy of these plants, as all are used by half or more of the cultural groups in British Columbia and adjacent portions of Washington and Alaska west of the Rocky Mountains. I would now like to turn to a detailed discussion of these plants. Sources for the data presented in Figures 2, 3, 4, 5, 6, 7, 8, 9 are listed in Table 4.

**Table 4:** Sources of Information for Figures 2–9

*Oplopanax horridus*, Figure 2	Sources
Tenaina, Tlingit, Kaigani Haida, Haida, Squamish, Cowichan/Halkomelem, Cowlitz, Okanagan-Colville, Tsimshian, Carrier, Thompson, Bella Coola, Kootenay, Sechelt, Mainland Comox, Ohiat Nootka, Nitinaht, Upriver Halkmomelem, Skagit, Sahaptin, Heiltsuq	[3,11]
Witsuwit'en	[25]
Haisla	(pers obs.), [29]
Cowlitz, Green River people, Skagit, Lummi	[30]
Sahaptin	[31]
Shuswap	[32]
Coast Salish	[33]
Kwakiutl	[34]
Crow, Cheyenne	[9]
Oweekeno	[9,35]
Tahltan	[35]
Makah	[9]
Tagish	[26]
Kaska	Pat Moore pers. com., LMJ 1999 fieldnotes
	
*Nuphar polysepalum *(*N. luteum *ssp. *polysepalum*), Figure 3	

Witsuwit'en	[25,46]
Carrier (non-use)	[46]
Tlingit	[36]
Haida	[37]
Thompson	[38]
Makah, Quinault	[30]
Ditidaht	[39]
Kwakiutl	[34]
Shuswap	[32]
Nuxalk	[40]
Okanagan-Colville	[41]
Inland Tlingit	[26]
Tagish	[26]
Kaska	LMJ fieldnotes 1998
Haisla, Henaksiala	[9]
Kitasoo	[9]
Upper Tenana	[9]
	
*Veratrum viride*, Figure 4	

Witsuwit'en	[25]
Carrier, Nisga'a	LMJ personal observations, fieldnotes
Tsimshian, Haisla, Lillooet, Bella Coola, Tlingit, Okanagan-Colville	[3]
Blood	[42]
Haisla	[29]
Thompson	[38]
Cowlitz, Quinault '*V. escholtzii*'	[30]
Bella Coola	[40]
Kwakiutl	[34]
Okanagan-Colville	[41]
Tagish	[26]
Inland Tlingit	[26]
Haida '*V. escholtzii'*	[37]
Kaska (identification not confirmed with specimen)	LMJ fieldnotes
Kutenai	[9]
Kitasoo	[9]
Owekeeno	[9]
Tsimshian	[9]
Haisla and Henaksiala, Henaksiala	[9]
Flathead	[9]
Blackfoot	[9]
	
*Abies lasiocarpa *[=*Abies balsamea *ssp. *lasiocarpa*], Figure 5	

Blackfoot	[42]
Witsuwit'en	[25]
Carrier	[43]
Thompson	[38]
Sahaptin	[31]
Shuswap	[32]?
Okanagan-Colville	[41]
Kaska	LMJ fieldnotes 1997–1999
Tagish	[26]
Inland Tlingit	[26]
Tahltan	LMJ fieldnotes 1987
Tlingit	[36]
	
*Abies amabilis*, Figure 5	

Ditidaht	[39]
Haisla	[44,29], LMJ fieldnotes
Nuxalk	[39]
	
*Abies grandis*, Figure 5	

Ditidaht	[39]
Coast Salish	[33]
Green River	[30]
Sahaptin	[31]
Shuswap	[32]
Kwakiutl	[34]
Okanagan-Colville	[33]
	
*P. lutzii*, hybrid spruce (*P. sitchensis, engelmannii *and *glauca*) and hybrids of *Picea engelmannii *and *P. glauca*, Figure 6:	

Witsuwit'en	[25]
Carrier – &*P. engelmanii *× *glauca*	[42] & [23,43]
Thompson (all three species)	[38]
Sahaptin	[31]
Okanagan-Colville	[41]
Chilcotin	[38]
Shuswap	[9]
Tahltan	LMJ and [35]
Sekani	[46]; LMJ fieldnotes 1999
Kaska	LMJ fieldnotes 1998
	
*Picea sitchensis*, Figure 6	

Haisla	[29]
Makah, Quinault	[30]
Nuxalk	[40]
Kwakiutl	[34]
Haida	[37]
Nuu-Chah-Nulth	Turner pers. comm. 1999
	
*Picea glauca*, Figure 6	

Upper Tenana	[9]
Tagish	[26]
Inland Tlingit	[26]
Tlingit	[36]
	
*Pinus contorta*, Figure 7	

Witsuwit'en	[25]
Blood, Cree	[42]
Thompson	[38]
Quinault	[30]
Sahaptin	[31]
Shuswap	[32]
Nuxalk	[40]
Kwakiutl	[34]
Tagish	[26]
Inland Tlingit	[26]
Kaska	LMJ fieldnotes 1997–1998
Tahltan	LMJ fieldnotes 1987
Carrier	[23.43]
Okanagan-Colville	[41]
Blackfoot; Carrier N; Carrier S; Flathead; Kutenai; Coast Salish; Sekani;Tlingit; Haisla and Henaksiala	[9]
Haida	[37]
	
*Juniperus communis*, Figure 8	

Witsuwit'en	[25]
Carrier	[45]
Cree	[42] & Siegfried pers. comm. 1994
Blackfoot	[42]
Thompson	[38]
Sahaptin	[31]
Shuswap	[32]
Nuxalk	[40]
Kwakiutl	[34]
Okanagan-Colville	[41]
Kaska	LMJ fieldnotes 1997–1998
Tahltan	LMJ interview notes 1987
Tagish	[26]
Inland Tlingit (as "crowberry")	[26]
Henaksiala	[9]
Upper Tenana	[9]
Haida	[37]
Coast Salish (Victoria area)	Turner pers. comm 1999
	
*Juniperus scopulorum*, Figure 8	

Thompson	[38]
Swinomish	[30]
Sahaptin	[31]
Shuswap	[32]
Okanagan-Colville	[41]
	
*Sambucus racemosa*, Figure 9	

Ditidaht	[39]
Thompson	[38]
Haisla	[29]
Makah, Cowlitz, Squaxin, Quinault	[30]
Coast Salish	[33]
Nuxalk	[40]
Kwakiutl	[34]
Witsuwit'en	LMJ notes
Carrier	[23,43]
Carrier Southern, Hesquiat, Menominee, Micmac, Okanogan, Sikani (*S. racemosa *ssp. *pubens *var. *arborescens*)	[9]
Cowlitz, Haisla and Henaksiala, Henaksiala, Hoh, Makah, Nitinaht, Quileute; Quinault; Squaxin	[9]
Haida	[37]

**Table 5:** List of Voucher Specimens on Deposit at Royal British Columbia Herbarium (V) and at University of Alberta Herbarium (ALTA)

**Royal BC Museum Herbarium [V]**	LMJ Gottesfeld collection numbers		
species	spec. #	date sent	Accession #
*Arctostaphylos uva-ursi* (L.) Spreng.	TM14	Oct-87	V159833
*Calla palustris *L.	TM11	Oct-87	V159830
*Dryopteris expansa *(K.B.Presl) Fraser-Jenkins & Jermy	Eth 29	Sep-88	V159855
*Dryopteris expansa *(K.B.Presl) Fraser-Jenkins & Jermy	Eth 30	Dec-88	V159383
*Geum macrophyllum *Willd.	Eth 38	Feb-90	V159101
*Heracleum lanatum *Michx.	TM19	Oct-87	V159826
*Juniperus communis *L.	TM16	Oct-87	V159829
*Nuphar polysepalum *Engelm.	TM12	Oct-87	V159831
*Oplopanax horridum *(Smith) Miq. (= Oplopanax horridus)	TM18	Oct-87	V159827
*Picea × Roche (= Lutzii)*	TM17	Oct-87	V159828
*Pinus contorta *Dougl.	TM15		V159822
*Prunus pensylvanica *L.	Eth 32	Dec-88	V159388
*Sambucus racemosa *L.	TM 20	Oct-87	V159825
*Shepherdia canadensis *(L.) Nutt.	TM22	Oct-87	V159823
*Sorbus scopulina *Greene	TM21	Oct-87	V159824
*Veratrum viride *Ait.	TM13	Oct-87	V159832
		record date	
*Abies amabilis *(Dougl.) Forbes	Eth 12	May-88	V159386
*Abies lasiocarpa*(Hook.) Nutt.	Eth 04	May-88	V159387
*Acer glabrum *(Torr.) var. *douglasii*(Hook.) Dippel	Eth 48	Nov-90	V162924
*Achillea millefolium *L.	Eth 74	Jun-94	V163181
*Allium cernuum *Roth	Eth 65	Jun-94	V162921
*Alnus crispa*(Drylander ex Ait.*) Pursh *ssp. *sinuata *(Regel) Hultén	Eth 44	Nov-90	V162931
*Alnus incana *(L) Moench ssp. *tenuifolia *(Nutt.) Breitung	Eth 45		V163192
*Alnus rubra *Bong.	Eth 42	Nov-90	V163185
*Amelanchier alnifolia *Nutt.	Eth 58	Jun-94	V162986
*Apocynum androsimaefolium *L.	Eth 34	Jul-89	V162632
*Apocynum androsimaefolium *L.	Eth 68	Jun-94	V162923
*Aralia nudicaulis *L.	Eth 81	Jun-94	V162920
*Arctostaphylos uva-ursi *L.	Eth 77	Jun-94	V163183
*Artemesia tilesii *Ledeb.	Eth 36	Jul-89	V162631
*Artemesia tilesii *Ledeb.	Eth 37	Jul-89	V162630
*Betula papyrifera *Marsh.	Eth 50	Nov-90	V162919
*Cornus stolonifera *Michx.	Eth 70	Jun-94	V163187
*Dryopteris expansa (*K.B.Presl) Fraser-Jenkins & Jermy	Eth 25	Sep-88	V159382
*Epilobium angustifolium *L.	Eth 52	Nov-90	V162925
*Equisetum arvense *L.	Eth 82	Jun-94	V163186
*Equisetum arvense *L.	Eth 89	Jun-95	
*Lonicera involucrata *(Rich.) Banks	Eth 43	Nov-90	V162927
*Picea mariana *(Mill.) Britt., Sterns & Pogg.	Eth 21	May-88	V162911
*Populus balsamifera *L. ssp. *trichocarpa *(Torr. & Gray) Hult.	Eth 40	Nov-90	V162928
*Populus tremuloides *Michx.	Eth 41	Jun-94	V163189
*Prunus pensylvanica *L.	Eth 86	Jun-95	
*Prunus virginiana *L.	Eth 87	Jun-95	
*Ribes oxyacanthoides *L.	Eth 57	Jun-94	V162930
*Rosa acicularis *Lindl.	Eth 75	Jun-94	V163182
*Rubus parviflorus *Nutt.	Eth 66	Jun-94	V162922
*Salix scouleriana *Barratt	Eth 49	Nov-90	V162929
*Sedum divergens *Wats.	Eth 51		V162918
*Sorbus scopulina *Greene	Eth 78	Jun-94	V163184
*Symphoricarpos albus *(L.) Blake	Eth 46	Nov-90	V162926
*Thuja plicata *Donn ex D. Don	Eth 09	May-88	V159384
*Tsuga heterophylla *(Raf.) Sarg.	Eth 13	May-88	V159385
*Urtica dioica *L.	Eth 53	Jul-91	V162985
*Vaccinium membranaceum *Dougl.	Eth 71	Jun-94	V163179
*Vaccinium ovalifolium *Smith	Eth 72	Jun-94	V163180
*Viburnum edule *(Michx.) Raf.	Eth 69	Jun-94	V163188
			
Leslie Main Johnson/LMJ Gottesfeld specimen numbers and accession numbers			
species	spec. #	record date	Accession #

**U of Alberta Cryptogamic Herbarium**			
*Fomes fomentarius*	Eth 85	Nov-94	
*Inonotus obliquus *(Pers:Fr.) Pilat	Eth 54	Feb-91	
			
**University of Alberta Herbarium [ALTA]**			ALTA
*Tsuga heterophylla *(Raf.) Sarg.	Eth 02	Jun-88	106827
*Thuja plicata *Donn ex D. Don	Eth 07	May-88	100809
*Salix discolor *Muhlenberg	Eth 23	May-88	
*Apocynum androsimaefolium *L.	Eth 35	Jul-89	
*Alnus crispa *(Drylander ex Ait.*) *Pursh ssp. *sinuata *(Regel) Hultén	Eth 55	Nov-90	
*Populus balsamifera *L. ssp. *trichocarpa *(Torr. & Gray) Hult.	Eth 56	Jun-94	
*Corylus cornuta *Marsh.	Eth 39	Jun-94	
*Urtica dioica *L.	Eth 60	Jun-94	
*Populus tremuloides *Michx.	Eth 61	Jun-94	
*Ribes oxyacanthoides *L.	Eth 64	Jun-94	
*Rubus parviflorus *Nutt.	Eth 67	Jun-94	
*Cornus stolonifera *Michx.	Eth 73	Jun-94	
*Vaccinium ovalifolium *Smith.	Eth 76	Jun-94	
*Sorbus scopulina *Greene	Eth 79	Jun-94	
*Tsuga heterophylla *(Raf.) Sarg.	Eth 83	Jun-94	
*Prunus virginiana *L.	Eth 88	Jun-95	

Assistant Curator Brij Kohli accessioned specimens with numbers between 100809–100877 in 1999 No more specific attribution of numbers to individual specimens was recorded Specimens with no listed ALTA accession numbers are in this group

### Analyses of specific plants

#### Devil's club

Devil's Club (*Oplopanax horridus *(S.) T.&G. ex Miq.) is the most frequently mentioned and widely used medicinal plant (Table 3). Twenty-nine consultants gave information about uses of devil's club. The most frequent reported use was as a tonic (7 consultants). Use of decoctions for respiratory illnesses, TB, arthritis treatment, heart or blood medicine, and purification or enhancement of luck were mentioned by four consultants each. Treatment of stomach ailments or ulcers, wounds or skin ulcers, and cancer were mentioned by 3 consultants each. Treatment of headache, flu or stomach flu, and use as a cleanser or purgative, as a cure-all or as a smudge were also mentioned.

The range of devil's club is restricted to western North America and coincides largely with the general culture area of the Northwest Coast and adjacent inland groups among which extensive pre-contact and present-day trading networks exist [10]. It is therefore unlikely that knowledge and use of devil's club was independently arrived at by other cultures. Figure 2 shows reported uses of devil's club among cultures within its range in western North America (see [11]for an extensive review of use of devil's club). Thirty-five groups west of the Rocky Mountains are reported to use devil's club (83%), while for seven groups there is no specific information available (17%). The degree of intercultural consensus on the value of devil's club is high. There are some reports of use of *Oplopanax japonicus *in Chinese medicine for treatment of cough and as an anti-pyretic [12], suggesting independent confirmation of some therapeutic effects of devil's club. *O. japonicus *is considered by Hultén [13] to be a subspecies of *Echinopanax horridum *(= *O. horridus*),

The available bioassay and phytochemical data also suggest the value of devil's club in treating various kinds of illnesses. Antibacterial and antimycobacterial properties have been demonstrated by McCutcheon and her colleagues [14,15]. They obtained particularly impressive results with devil's club against *Mycobacterium tuberculosis *and *M. avium*, obtaining complete inhibition of both species with 20 mg of extract. This lends strong support to the ethnomedical use of devil's club for tuberculosis treatment. Kobaisy *et al*. [16] have isolated four polyynes from devils club bark which showed strong antimycobacterial activity. McCutcheon and her colleagues also found antiviral effects against respiratory syncytial virus [17]. Old clinical studies from the 1930's give some indication of possible hypoglycemic properties, suggesting efficacy in regulating blood sugar in diabetic patients, but other clinical studies do not confirm the early results. A more recent Russian study [18] shows effective experimental reduction of blood glucose in diabetic rats by a tincture of *Oplopanax japonicus*.

A recent study by Blaxton *et al*. [19] discusses phytochemicals present in *Oplopanax horridus *bark extract, and the clinical properties of some compounds which support the ethnomedical uses. Four sesquiterpenes were isolated (alpha cubebene, trans-nerolidol, spathulinol, and oplopanone) by Blaxton and his colleagues. Transnerolidol has shown anti-colon cancer effects in rats (Wattenburg 1991 cited in [19]), and is reported to be spasmolytic in mice (Budavari 1989 cited in [19]). Stigmasterol and beta sitosterol have been reported to have antirheumatic and anticholesterolemic properties and oplopanone has been used for antitussive and antipyretic properties (Budavari 1989 cited in [19]).

### Use of yellow pond lily

Yellow pond lily(*Nuphar polysepalum *Engelm., sometimes classified as *Nuphar luteum *(L.) Sibth. & Sm. ssp. *polysepalum*(Engelm.) Beal), was mentioned by 17 consultants. Nineteen cultural groups within the range of this species north of Oregon use pondlily for medicine, while for 2 groups recorded information does not indicate use for medicine (Figure 3). Six Gitksan consultants reported use of the rhizome for TB; four reported its use as a poultice for fractures, three as a poultice for arthritis, and three for poulticing sores and wounds. Two modern consultants, and one archival source report use of the rhizome for a male contraceptive. Use of pondlily rhizome for "sickness", as an appetite stimulant, for liver medicine, and for aches and pains were also mentioned.

Pond lily showed moderate activity against *Mycobacterium tuberculosis *in vitro, achieving complete inhibition at concentrations of 100 mg [15]. It was often mixed with devil's club in antitubercular preparations, which makes efficacy very likely, especially when taken over prolonged periods of time as modern anti-tubercular medicines are. The properties of alkaloids known to occur in the rhizome of *Nuphar luteum *cannot be neatly correlated with Gitksan ethnomedical uses, although antispasmodic properties of nupharine might aid appetite by suppressing nausea. Other Gitksan uses of yellow pond lily rhizome suggest possible antiinflammatory or analgesic properties, but high levels of steroidal or other known antiinflammatory compounds or analgesics have not been reported for *Nuphar luteum*. Zhang *et al*. [20] have suggested that immune suppressant properties of deoxynupharidine, which occurs in *N. variegatum *as well as the Chinese *N. pumilum*, may account for the use of *Nuphar pumilum *in Chinese folk medicine for rheumatoid arthritis, as rheumatoid arthritis is an autoimmune disease.

### Indian hellebore

Indian hellebore (*Veratrum viride *Ait.) rhizome was mentioned by 13 consultants in recorded interviews, and in various other (unrecorded) conversations, suggesting its widespread collection and use at present Twenty-four other cultures in northwestern North America are reported to have used *Veratrum viride *for medicine, while one culture (Sahaptin) was reported not to use *Veratrum viride *rhizome (Fig. 4).

Use of *Veratrum viride *for purification or as a smudge was mentioned by 9 consultants. Smudges are used as treatment for stroke or mental illness, to counteract evil magic, and to repel ghosts or evil spirits. Use for hunting luck was mentioned by 5 people. Five people made reference the practice of carrying a piece of the dried rhizome for protection from bad luck or evil magic. Three consultants described bathing in an infusion of hellebore rhizome as an external cleanser. Treating skin and hair was mentioned by two people. Two people also described use of Indian hellebore as a "germ killer", which can be put in wash water to cleanse laundry of evil influences, vermin or germs (in the medical sense of bacteria or viruses). Two consultants mentioned use as a snuff to clear the sinuses. (I can verify from observation that it induces dramatic sneezing and running of the nose when taken this way). Uses as a nightmare remedy and as a sleepwalking remedy were also mentioned.

Many of the uses of *Veratrum viride *are clearly related to cultural notions of spiritual potency of plants, and concepts of cleansing incorporate notions of spiritual as well as physical cleanliness. It is possible that salience, toxicity and/or conspicuous thorniness (as with devil's club, also seen as spiritually powerful and cleansing) contribute to these concepts of potency. The toxicity and potential for loss of life caused by careless use of *Veratrum *are certainly understood locally and could contribute to its widespread perception as a powerful and dangerous plant. Notions of "dirt" or "contamination" can accommodate to contemporary notions of disease causation by germs, but the local concepts also relate to notions of spiritual as well as physical cleanliness or "dirt". These notions may explain the concept of Indian hellebore as a germ killer; potential insecticidal properties could also be involved in cleaning of laundry.

Most of the known active compounds in *Veratrum viride *are reported from the roots rather than the rhizomes [21]. Gitksan use is primarily of the rhizomes, rather than the attached fleshy roots, though some use is also made of the roots, which may be left attached to rhizome pieces when they are dried. Ethnomedical uses for topical analgesia and skin treatment are confirmed by known chemicals, at least in the roots. The toxicity of *Veratrum *is recognized by the Gitksan, who do not use it internally. They do not make use of the alkaloids to lower blood pressure; these form the basis of the use of *Veratrum viride *in modern pharmacology, as a base for synthesis of hypotensive medications [22].

### Conifer barks and pitches

Conifer barks and pitches are still widely collected. Uses of *Picea *and *Pinus*, and *Abies*, were mentioned by 9 consultants each (Table 3). The liquid and solid pitches of spruce, pine, and fir are used for wound dressings and taken internally. All three species, and other related species, are widely used in northwestern North America. Thirteen groups in the map area are known to use *Abies lasiocarpa *(Hook.) Nutt., while 8 use *Abies grandis *(Dougl.) Forbes or *Abies amabilis *(Dougl.) Forbes (Fig. 5). For 11 groups there is no record of use of *Abies*.

As the spruce used by the Gitksan *Picea *× *lutzii*, or Roche spruce, is a hybrid swarm of *Picea engelmannii *Parry and *P. glauca *(Moench) Voss with *P. sitchensis *(Bong.) Carr, I have mapped use of all three of these species in northwestern North America (Fig. 6). Seventeen groups are known to use these species for medicines, while for nine groups there are no reports in the literature of medicinal use.

Altogether, 16 groups are reported to use *Pinus contorta *Dougl. for medicine, while 21 cultures are not reported to use lodgepole pine (Fig. 7) (but many of the more southern groups do use other species of pine such as ponderosa or western white pine).

*Abies *is used for a variety of purposes: the bark in mixed decoctions for treatment of tuberculosis, or as a tonic, or for internal medicine or lung disease (Additional file 1). The liquid pitch is regarded as the strongest part of the subalpine fir, and is used for TB treatment, as a purgative or cleanser, or for cuts or boils. Pine and spruce pitch and bark have been given together, as consultants often consider them interchangeable. There is high consensus on use of pitch of pine or spruce for infections, burns and boils (9 of 9). Other uses are in tonic "wood medicine" mixed decoctions and for treatment of tuberculosis.

Conifer pitch has antibiotic properties, especially spruce pitch, as Ritch-Krc *et al*. [23] showed in their research, where they obtained very strong inhibition of bacteria and *Candida *with the pitch of *Picea glauca *from central British Columbia, and moderately strong inhibition of bacteria with pitch from *Pinus contorta*. Interestingly, their assay results found no significant inhibition of bacteria or *Candida *by *Abies *pitch, which is generally rated a better medicine by indigenous consultants. However, these negative results could have been influenced by technical difficulties mixing pitch with the growth medium (Turner, personal communication 1999).

### Common juniper

Common juniper, *Juniperus communis *L., is widely used for medicine around the world. Juniper is called "boughs of the supernatural" or **laxsa laxnok**in Gitksan (Fig. 8). Eight consultants mentioned use of juniper boughs for medicine. There were 17 records of use of common juniper in the map area (and 6 records from Eastern North America and 1 from the western Canadian Arctic), and 15 cultural groups for which there was no record of use. There were no records of non-use of juniper. Five consultants used mixed decoctions containing juniper for respiratory illnesses, two for tonics, and 2 used juniper boughs as a smudge for spiritual purposes

A detailed discussion of the many active compounds found in juniper and their diverse effects is beyond the scope of this paper. Extracts of *J. communis *showed moderately strong bacterial inhibition of all species tested [14]. Numerous active chemicals are found in "berries" and other parts of the plant, including-umbelliferone, camphene, camphor, myrcene, delta-3-carene ("berries"-), alpha-pinene and limonene, rutin, borneol, citronellol, and umbelliferone. These compounds exhibit diverse activities, including: antiseptic, bactericidal, fungicidal and antiviral properties; analgesic, anesthetic, antihistiminic, antiinflammatory, expectorant and antitussive properties; spasmolytic, spasmogenic and sedative properties; antiedemic, antidiabetic, anticancer and cancer preventative, antiatherogenic, and liver protective actions; and reduction of fever [21]. In addition, limonene, alpha-pinene and rutin are also known to be anti-nephritic or nephrotoxic, which corresponds to the known kidney damaging effects which *J. communis *extracts can produce (Oates pers. comm 1987).

### Red elderberry

Red elderberry, *Sambucus racemosa *L., is the last medicine mentioned by 5 or more consultants. It is a strong emetic. Perhaps it was mentioned by 5 consultants because of the dramatic and memorable nature of its reported effects! In Gitksan thought dirt or contamination, at both spiritual and physical levels, are implicated in disease causation. Therefore, cleansing with an emetic preparation from elderberry root bark was seen as an effective treatment for a serious ailment like influenza. The other reported use is as part of a spiritual smudge medication. Thirteen cultural groups in the map area used red elderberry as medicine, while there was no record of use for 13 additional cultural groups (Fig. 9). Use of roots or stem inner bark as an emetic was recorded by 7 of 22 other groups which use red elderberry for medicine [9], sources in [2]. Elderberry species contain cyanogenic glycosides in roots, stems and leaves, and an unidentified cathartic compound in the bark and roots ([24]: 148).

## Discussion

### The movement of medicinal plant knowledge and Use

Trade and diffusion of knowledge occur despite significant linguistic differences. In some cases the name may diffuse across language boundaries, reinforcing the concept that the knowledge of use may also have diffused. An example of this is the name for yellow pond lily **gahldaats **in Gitksan, which is a loan word from the Witsuwit'en (Athapaskan) word, **këlht'ats **[25]. Uses among the two groups are similar, and indeed, individual users may be both bilingual and bicultural.

Recent documented examples of knowledge diffusion across cultural boundaries include anecdotal acknowledgement of learning of a particular use of plants by members of another cultural group: one consultant used soapberries as arthritis medicine; he said he learned this "from the Coast people". Another example includes use of the lichen lungwort (*Lobaria pulmonaria*); use of this medicine was reputedly taught to the my colleague's great uncle by a healer from Port Simpson, a Coast Tsimshian community. The use of devil's club for diabetes treatment by an elder from Gitsigyukwhla was reportedly learned from someone from Hartley Bay, another Coast Tsimshian community. In the southern Yukon, Catherine McClellan documented that use of Indian hellebore by Tagish people was learned from contact with people on the Coast [Tlingit] ([26]:226).

Finally, the trade of medicinal plants may diffuse knowledge even beyond the range of occurrence of the plant. The Kaska in the Southern Yukon apparently obtain devil's club inner bark by trade, and have incorporated its use in their medicinal plant repertoire. The Tagish also have to travel over the mountains or trade for devil's club [26]. Going in the other direction, various Gitksan people like "caribou leaves" (*Artemisia tilesii *Ledeb. ssp. *elatior *(Torr. & Gray) Hult.), the use of which they have recently learned from Tahltan and Kaska people in the Telegraph Creek/Dease Lake areas of northern British Columbia. Some people either travel by car to the Stikine River drainage to harvest a supply or to trade for it.

The modern use of sweetgrass (*Hierochloe odorata *(L.) Beauv.) and sage (*Artemisia *spp.) for smudging is another such example; the trade network for obtaining sweetgrass is extensive, and modern use is learned from various Cree and other healers from Alberta. Modern sage use seems to come both from the southern interior of British Columbia and from Alberta and the Plains and is probably spread both by the pow-wow circuit and by sweats and healing ceremonies.

In light of evidence of extensive trade, both in the past and at the present time, I therefore cannot assume that knowledge and use of medicinal plants has been arrived at independently in cultures of British Columbia and adjacent Alaska and Washington. Use of "intercultural consensus" is another way to indicate the increased likelihood of empirical efficacy of plants widely used by a number of different cultural groups.

Careful consideration needs to be given to the conditions under which widespread trade and contact between groups across language boundaries suggests that intercultural consensus in medicinal plant uses should be examined. One of the most obvious issues for a map-based method is, how do you decide what the boundaries of the map area should be? In the case of the Northwest Coast and adjacent interior areas of British Columbia, the southern Yukon, and Washington State, geography and biogeography help to define the area of interest. I think this kind of analysis will have to proceed on a specific region-by-region basis.

### Ethnomedical concepts in Gitksan healing

Aside from concepts like spiritual cleansing, luck bringing and protection, which cannot be evaluated in biomedical terms, there are several aspects of Gitksan concepts of healing which do affect choices of plant therapies and how they are used. For example, Gitksan people believe that illness must be treated holistically. This means that food and self-restraint or control will be part of the recovery process, and that taking medicines will be incorporated into a healthy lifestyle, as in the prolonged use of tonics. Gitksan in general expect to take medicines over a relatively long period of time (ranging from weeks to months, depending on the nature of the illness), in order for them to be effective. Furthermore, I was often told that one should take a medicine "whenever you feel thirsty", in effect, to replace intake of pure water with intake of medicine, to immerse oneself in therapy.

Another aspect of Gitksan treatment is that dirt or contamination, at both spiritual and physical levels, are implicated in disease causation. Therefore, cleansing (purgative or emetic herbs) may be employed to treat a variety of ailments; the clearest example is the treatment of influenza or inability to eat with a powerful emetic, in order to leave the digestive tract clean and ready to receive healing nourishment. From a biobehavioral perspective one would have to rate use of elderberry root as an emetic to be an effective treatment for influenza and serious sickness; the emetic effects are confirmed by other groups, and by clinical reports of purgative chemicals in the bark and roots of elderberry species. The congruence of the Gitksan and biomedical understandings would not be as great; physicians do not usually consider emesis or purgation to be necessary for recovery from viral illnesses.

### The "negative evidence"

Thus far, I have documented a neat fit between ethnomedical use of plants and potential or known phytochemicals with physiological effects. In the course of the literature search to confirm Gitksan use of specific plants by finding other groups using various plants and to locate information on phytochemicals and activities of plants used by the Gitksan, I could not help but notice multiple records of use and active phytochemicals or assays of plants found in Northwest British Columbia which are not used by the Gitksan. In total, some 70 plants present in the Gitksan territories which are used medicinally by other groups are not known to have been used by the Gitksan. Some of these have a number of active phytochemicals and even clinical studies supporting their efficacy. Additional file 2 summarizes known properties of phytochemicals in the following two plants which are easily available in Gitksan territory, but not used for medicinal purposes by Gitksan people as far as I have been able to discover.

Kinnikinnick (*Arctostaphylos uva-ursi *(L.) Spreng.) is widely used in phytotherapy and herbology for treatment of urinary tract infections [27]; it also contains substantial amounts of arbutin [21]. The Gitksan, however, only collected the fruits for winter storage, but made no medicinal use of the plant.

Wild mint (*Mentha arvensis *L.) is used in many healing traditions around the world and contains many active phytochemicals, including menthol, in relatively large amounts (Additional file 2). It is highly valued by Kaska elders, for example, from whom I learned its uses after only a short period of fieldwork with a few elders. However, Gitksan elders to whom I described or showed the plant did not recognize the plant, and did not use it.

Although rather problematic for a naive empiricist model of medicinal plant use, the plants not used for medicine provide information about cultural selectivity. Many factors might be responsible for their non-use, ranging from the purely stochastic, to symbolic or contextual. One possible approach to understanding some of this variation would be through use of methods used for elucidating diet breadth in optimal foraging analyses. Some people have suggested that an erosion of ethnomedical and ethnobotanical knowledge might be responsible for these seeming gaps in the use of available and active potential medicinal plants. While this may explain individual instances, I find it unsatisfying as an explanation for such a large number of unused plants. Finally, a seminal paper by Glenn Shepard on Amazonian medicinal plant use suggests that cultural factors that create strongly contrasting ethnomedical models of the nature of illness and modes of healing may cause adjacent groups within a broadly similar ecological context to make quite different choices of medicinal plants to use in treating illness, and may dictate quite dissimilar modes of application or use [28].

### Limitations of comparative analysis as a confirmation of plant use efficacy

My analysis was limited by the quality of the data. Both the completeness of my own data, and the extent of recorded medicinal plant knowledge of other groups within the map area make quantitative analysis of intercultural consensus impossible. The medicinal plant knowledge of some cultures is relatively well described, while for other groups, little reliable documentation of plant use is available. Another difficulty is presented by judging what kinds of uses are similar, given a vast range of descriptions of ailments and uses, driven both by cross-cultural translation of symptomology and disease causation, and historical changes in our understandings within western culture. A recent concern by indigenous peoples regarding the appropriation of their medicinal plant knowledge means that some groups prefer to keep their healing knowledge private, limiting the possibilities of comparison.

The non-uniformity of healing plant knowledge within societies between specialist healers and lay people, and between men and women, compounds the difficulties of obtaining a relatively complete sample of healing plants used for any one culture. I have attempted to overcome this in my own data by consulting with a number of different men and women from several villages. However, none of my consultants were publicly acknowledged herbal healing specialists, and many people expressed the concern that much healing knowledge had already passed on with the Elders. Recorded data from other cultures may be based on many fewer consultants, and botanical identifications in older work may be questionable.

These caveats also apply more generally, both to the "negative evidence" of plants not apparently used by the Gitksan, and to plants used by the Gitksan for which no confirming records of similar use by other cultures can be found. Sometimes even when only one culture appears to have healing plant knowledge, the plant in question may still be known to contain phytochemicals that make it likely to be therapeutic for the conditions it is used for from the perspective of a physiologically based analysis. This may be the case for the use of red osier dogwood (*Cornus stolonifera *Michx.) to treat fractures, a use not reported elsewhere in the literature.

## Conclusion

In summary, consultant consensus, intercultural consensus, and evidence of biological activity all converge to support the likely efficacy of the eight most frequently mentioned medicinal plants used by the Gitksan. It is my contention that plants found to be efficaceous in healing, within the framework of local understandings of health and illness, will be retained in local ethnomedical tradition. One of the interesting results of the study is demonstration of the richness and diversity of individual healing knowledge and practice within Gitksan society. Limitations in the completeness of data, both for the Gitksan and for other cultural groups, make a rigorous quantitative analysis of intercultural consensus impossible at this time. Similarly, although indications of similar use by nearby or distant other cultural groups supports the likely efficacy of traditional herbal remedies, lack of such confirmation does not necessarily rule out effectiveness. Some factor of cultural selectivity seems to be responsible for the lack of recorded Gitksan use of other medicinal plants which are widely used and known to contain active phytochemicals, though random variations in knowledge, sampling error and erosion of traditional knowledge may also be implicated in the lack of records of use of certain plants. Assuming that the non-use is genuine, and not an artifact of research methods and contexts, many factors might be responsible for their non-use, ranging from the purely stochastic, to symbolic or contextual. Ethnomedical models must be taken into account, as well as the availability of other remedies which might produce a similar effect to the unused plant.

## Competing interests

The author(s) declare that they have no competing interests.

## Authors' contributions

This is a sole authored paper.

## Supplementary Material

Additional file 1 Analysis of Empirical Confirmation of Physiologically Based Ethnomedical Uses of Selected Frequently Mentioned Gitksan Medicinal Plants Click here for file

Additional file 2 Phytochemicals and Activities of Two Medicinal Plants Occurring in Northwest British Columbia Which Are Not Utilized by the Gitksan. Click here for file
