# Supplementary material for: Gitksan medicinal plants-cultural choice and efficacy
Source: J Ethnobiol Ethnomed. 2006 Jun 21;2:29. doi: 10.1186/1746-4269-2-29 (PMC1564001; doi:10.1186/1746-4269-2-29)
Supplement: Additional file 1 — Analysis of Empirical Confirmation of Physiologically Based Ethnomedical Uses of Selected Frequently Mentioned Gitksan Medicinal Plants [file 1746-4269-2-29-S1.pdf]

Analysis of Empirical Confirmation of Physiologically Based Ethnomedical Uses of Selected Frequently Mentioned Gitksan Medicinal Plants.

–Devil's Club *Oplopanax horridus*

•RESPIRATORY ILLNESSES: antibacterial activity demonstrated for methanol extracts of the bark; strong antimycobacterial activity against *Mycobacterium tuberculosis* and *M. avium* weaker anti-viral activity against respiratory syncytial virus [15,17]

•PURGATIVE: no information

•DIURETIC: no information

•STOMACH AILMENTS: antibacterial activity against *Salmonella typhimurium*

•WOUND DRESSING: antibacterial properties

• SKIN WASH AND SCENT REMOVER: likely to prevent bacterial skin conditions

•DIABETES: clinical evidence of hypoglycaemic effects of devil's club extracts, but other clinical studies failed to support these findings; Experimental support for hypoglycemic effects in experimentally induced diabetes in rats [18]

•CANCER: no known cancer-preventive or tumor-inhibiting chemicals

•TONIC: antibacterial and anti-viral properties might help prevent diseases

•ARTHRITIS: no known antiinflammatory or analgesic compounds

Empirical Confirmation:

Respiratory illness: Level 2 or 3 (16/36 groups)
Purgative: Level 1 (7/36 groups)
Diuretic: Level ?1 (1/36 groups-high blood pressure)
Stomach: Level 1 (3?/36 groups)
Wound dressing: Level 1 (5/36 groups)
Skin Wash: Level 1(4/36 groups)
Diabetes:Level 1 or 4? (5/36 groups)
Cancer prevention or early treatment: Level 1(4/36groups)
Tonic: Level 1 (7/36 groups)
Arthritis: Level 1 (15/36 groups)

–Yellow Pond Lily, *Nuphar polysepalum*

•TUBERCULOSIS- strong antibacterial activity demonstrated in methanol extracts by McCutcheon *et al* 1992; 1997: moderate mycobacterial activity against *Mycobacterium tuberculosis*.

•FRACTURES, SORES, SWELLING: no chemicals which are known to have antiinflammatory or or analgesic or other relevant properties are known to occur in *N.polysepalum* although relatively high levels of ß-sitosterol, which is a steroid, are known to occur in *Nymphaea odorata*, the white water lily. Deoxynupharidine has immune suppressant properties which could reduce inflammation in rheumatoid arthritis by reducing autoimmune reactions [20].

•APPETITE STIMULANT: none of the chemicals known to occur would affect appetite directly, although spasmolytic properties shown by nupharine might reduce nausea and therefore promote desire to eat.

•CONTRACEPTION: although this use was not validated by other groups, male contraception might be effective if sufficient ß-sitosterol were present in *Nuphar* rhizomes. This potential use cannot be validated until better characterization of *N. polysepalum* rhizomes is undertaken.

Empirical Confirmation

Tuberculosis medicine: Level 3 (7/17 groups show this use)
Poultice for fractures, sores, swellings: Level 1 (2?) (7/14 groups show this use)
Appetite Stimulant: Level 1 (1/17 groups used it for stomach ulcers and 1/17 for gallstones)
Contraception: Level 1* (1 or 2?/17-1 woman's medicine, 1 not independent)

–Indian or False Hellebore *Veratrum viride* (Physiologically Based Ethnomedical Uses)

•SKIN AILMENTS: anti-louse and fungicidal properties (roots)
•BOILS AND SWELLINGS: analgesic and counterirritant effects (roots); rhizomes have fewer known active chemicals
•ACHES AND PAINS (TOPICAL): analgesic and counterirritant chemicals (roots)
•LUNG HEMORRHAGE (topical, mixed poultice): a mixed poultice has properties deriving from all its components; unclear how Indian hellebore might stop hemorrhage unless compounds which lower blood pressure are absorbed transdermally and might lessen the bleeding.
Empirical Confirmation:
Skin ailments: Level 1 or 3 (7/27 groups)
Boils and Swellings: Level 2 (4/27 groups)
Aches and pains (topical): Level 2 (11/27 groups)
Lung hemorrhage (mixed, topical): Level 1 (3/27 groups)
Sinus decongestant: Level 1 (11/27 groups)

–Subalpine Fir *Abies lasiocarpa*
•CLEANSER: no known purgative properties
•WOUND DRESSING: tannins, astringent; bornyl acetate has antibacterial action
Abies spp. from Siberia show moderate-strong antibacterial activity against all species surveyed; results from *Abies lasiocarpa lasiocarpa* reported by Ritch-Krc *et al.* [23] confirm these results only weakly
•ARTHRITIS-no known active chemicals or activities
•TONIC-probable antiviral activity and antibacterial activity from bornyl acetate; Siberian species antibacterial activity
•RESPIRATORY ILLNESS-probable antiviral activity and antibacterial activity from bornyl acetate; Siberian species antibacterial activity
Empirical Confirmation
Cleanser: Level 1 (4/12-including bark decoction or pitch)
Wound dressing: Level 3 (5/12)
Respiratory illness: Level 3 (11/12)
Tonic: Level 2 (3/12)
Arthritis: Level 1? (2/12, but external use)

–Spruce *Picea* x *lutzii*(hybrid swarm of *P. sitchensis*, *P. engelmannii*, and *P. glauca*):
•TONIC, RESPIRATORY ILLNESS, WOUND DRESSING: strong bacterial inhibition and strong antifungal activity against *Candida albicans* and *Aspergillis fumigatus* [23].
•BURNS: tannins, which have topical astringent properties
Empirical Confirmation
Tonic-Level 2 (8/36 including blood tonic and general sickness)
Respiratory illness: Level 2 OR 3 (15/36)
Wound dressing: Level 2 OR 3 (13/36)
Burns:Level 1 (1/36, powdered needles as dressing)

–Lodgepole Pine (*Pinus contorta*)
•COLDS AND RESPIRATORY ILLNESS: moderate anti-bacterial activity (bark extracts; [14]; pitch [23]). Limonene is sedative and spasmolytic, antiviral, "antipharyngitic",and quercetin is antihistamine. Tips for colds-quercetin, isorhamnetic are antihistaminic, antiinflammatory and spasmolytic.
•WOUNDS, SORES, BURNS: bark-anti-bacterial activity; quercetin (bark) is anti-herpes, antihistaminic and antidermatitic; limonene (oleoresins) is bactericidal and virucidal
•"SICKNESS": bark extracts are antibacterial, alpha-pinene, limonene and quercetin are antiflu, and quercetin and bornyl acetate are anti-viral
Empirical Confirmation
Colds and respiratory illness:Level 3 (15/24)
Wounds, sores, and burns: Level 3 (12/24, possibly +1 for syphillis)
"Sickness": Level 2 (5/24, including flu and "weakness")

–Juniper, *Juniperus communis*
•SMUDGE: various compounds used in perfumery; use for spiritual and non-material purposes. Use confirmed by 1/27 other groups.
•RESPIRATORY ILLNESS: extracts of *J. communis* showed moderately strong bacterial inhibition of all species tested [14]. Numerous active chemicals are found in "berries" and other parts of the plant, including-umbelliferone (antihistaminic, antiseptic, fungicide), camphene (spasmogenic), camphor (analgesic, anesthetic, aexpectorant, antitussive, antihistaminic, myrcene (bactericidal), delta-3-carene ("berries"-antiinflammatory and bactericidal), alpha-pinene and limonene (antiviral, influenza virus), rutin (antiviral), borneol (analgesic, antiinflamatory, febrifuge, spasmolytic),and citronellol (bactericidal, sedative); limonene, alpha-pinene and rutin are also known to be anti-nephritic or nephrotoxic, which corresponds to the known kidney damaging effects which *J. communis* extracts can produce (Oates pers. comm 1987).
•TONIC: in addition to the effects shown above, umbelliferone has colorectic, fungicidal, lipoxygenase inhibitory properties; camphor is antifibrinositic; rutin is antiedemic, antidiabetic, anticancer and cancer preventative, antiatherogenic, and hepatoprotectant; citronellol is candidicidal, and fungicidal, which would have diverse health-promoting effects.
Empirical Confirmation
Smudge: NA (6/34)
Respiratory illness:Level 3 (17/34)
Tonic: Level 3 (7/34)
