# Supplementary material for: Gitksan medicinal plants-cultural choice and efficacy
Source: J Ethnobiol Ethnomed. 2006 Jun 21;2:29. doi: 10.1186/1746-4269-2-29 (PMC1564001; doi:10.1186/1746-4269-2-29)
Supplement: Additional file 2 — Phytochemicals and Activities of Two Medicinal Plants Occurring in Northwest British Columbia Which Are Not Utilized by the Gitksan. [file 1746-4269-2-29-S2.pdf]

Phytochemicals and Activities of Two Medicinal Plants Occurring in Northwest British Columbia Which Are Not Utilized by the Gitksan.

Potential clinically useful properties are indicated by underlining; potentially toxic or undesirable activities are shown by use of *italics*. Hormonal activities affecting reproduction shown with double underline. Properties whose clinical relevance is not clear to me are left unmarked. Other properties (shown in parentheses). Properties taken from [21] .

Arctostaphylos uva-ursi
Kinnikinnik or bearberry

allantoin<br>plant	antiinflammatory, antipeptic, antipsoriac, antiulcer, immunostimulant, keratolytic, superative, vulnerary
arbutin<br>5-12% leaf	antiseptic, antitussive, artemicide, bactericide, candidicide, diuretic, insulin sparing, (pesticide), urinary-antiseptic
alpha-amyrin	antitumor, *cytotoxic*
ß-sitosterol	androgenic, *anorexic*, antiadenomic, antiandrogenic, *antifeedant*, antifertility, antigonadotrophic, antiinflammatory, antileukemic, antimutagenic, antiprogestational, antiprotastadenomic, antiprostatiitic, antitumor, antiviral, artemicide, bactericide, cancer-preventative, candidicide, estrogenic, gonadotrophic, hepatoprotective, hyopcholesterolemic, hypoglycemic, hypolipedemic, (pesticide), spermicide, viricide
betulinic acid	antitumor, *cytotoxic*
citric acid<br>leaf	antiaphthic, anticalculic, anticoagulant, antioxidant, antitumor, disinfectant, hemostat, litholytic, refrigerant
formic acid<br>leaf	antiseptic, antisyncopic, counterirritant, (pesticide)
gallic acid<br>plant	ACE inhibitor, anticarcinomic, antifibrinolytic, antioxidant, antiseptic, antiviral, astringent, bacteristatic, cancer-preventative, but *carcinogenic*, hemostat, *nephrotoxic*, (pesticide), styptic
hyperin<br>leaf	antiinflammatory, antioxidant, antitussive, antiviral, capillary fortifier, capillarigenic, diuretic, hepatoprotective, hypotensive, (pesticide), viricide
isoquercitrin<br>leaf	*antifeedant*, cancer-preventative, capillarigenic, diuretic, hypotensive, (pesticide)
lupeol<br>plant	antitumor, antiurethrotic, *cytotoxic*
malic acid<br>leaf	bacteristat, bruchiphobe, hemopoietic, (pesticide), sialagogue
monotropein<br>leaf	cathartic
myricetin	*antifeedant*, *antigastric*, antiinflammatory, cancer

leaf	preventative, diuretic, (larvistat and pesticide)
oleanolic-acid<br>plant	*abortifacient*, anticariogenic, antifertility, antihepatotoxic, antisarcomic, cancer-preventative, cardiotonic, diuretic, hepatoprotective, uterotonic
quercetin<br>leaf	5-lipoxygenase inhibitor, aldose-reductase inhibitor, (allelochemic), anti-tumor promotor, anti-Crohn's, anti-PMS, antiaggregant, antiallergic, antianalhylactic, antiasthmatic, anticataract, anticolitic, antidermatitic, antidiabetic, antiestrogenic, *antifeedant*, antiflu, anti-rabies, antiherpetic, antipolio, antiviral, HIV reverse transcriptase inhibitor, *antigastric*, antihepatotoxic, antihistaminic and antiinflammatory, antileukotrienic, antilipoperoxidant, antioxidant, antiperiodontal and antiplaque, antipermeability, antipharyngitic, antipodriac, antisporiac, antitumor, antiradicular, bactericide, calmodulin-antagonist, cancer preventative, protects capillaries, cyclooxygenase-inhibitor, *cytotoxic*, hypoglycemic, insulinogenic, (juvabional, larvistat, pesticide), lipoxygenase inhibitor,mast-cell stabilizer, *mutagenic*, spasmolytic, *teratologic*, *tumorigenic*, vasodilator, xanthine oxidase inhibitor, CAMP phosphodiesterase inhibitor
quinic acid	choleretic
tannin<br>6-20% bark	antidiarrheic, antidysenteric, antimutagenic, anti-nephritic, antioxidant, antiradicular, antiviral, bactericide, cancer-preventative, hepatoprotective, (pesticide), *psychotropic*, viricide.
ursolic acid	antidiabetic, antiinflammatory, antileukemic, antiobesity?, antitumor, *CNS depressant*, cancer-preventative, *cytotoxic*, diuretic, hepatoprotective, hypoglycemic, (piscicide)
uvaol	antitumor, *cytotoxic*

Mentha arvensis
wild mint, field mint

1,8-cineole	(allelopathic) anesthetic, antibronchitic, anticatarrh, antilaryngitic, antipharyngitic, antirhinitic, antiseptic, antitussive, bactericide, CNS-stimulant, choleretic, counterirritant, dentifrice, expectorant, fungicide, liver toner, hypotensive, (insect repellant and pesticide), rubifacient, sedative
acetic acid<br>plant	acidulant, antiototic, antivaginitic, bactericide, expectorant, fungicide, mucolytic, *osteolytic*, (pesticide), protistacide, *spermicide, ulcerogenic*, verrucolytic
alpha-pinene<br>plant 10-300 ppm	antiinflammatory, cancer-preventative (coleoptiphile, insectifuge, insectiphile, pesticide)
camphene	*spasmogenic*
carvone<br>leaf<br>0.81-2.6%	antiseptic, CNS stimulant, cancer-preventative, carminative (insecticide, insect repellant, pesticide) vermicide

caryophyllene<br>plant	antiedemic, antiinflammatory (insect repellant, perfumery, pesticide, termitifuge) spasmolytic
eugenol<br>essential oil	analgesic, anaesthetic, antiaggregant, antiedemic, *antifeedant*, antiinflammatory, antioxicant, antiprostaglandin, antiseptic, antiulcer, cancer-preventative, candidicide,choleretic, *cytotoxic*, febrifuge, fungicide, (apifuge, insectifuge, juvabional, larvicide, pesticide, herbicide), *irritant, ulcerogenic* , vermifuge
formic acid<br>plant	antiseptic, antisyncopic, counterirritant, (pesticide)
furfural<br>plant 2-5ppm	antiseptic, fungicide (insecticide, pesticide)
hesperidin<br>plant	*antiDNA, antiRNA*, antiallerginic, antioxidant, antistomatitic, antiviral, capillariportective, choleretic (pesticide), vasopressor
limonene<br>leaf<br>20-3520 ppm	acetylcholinesterase inhibitor--anti-Alzheimerian?, anticancer, antilithic, antiviral, viricide, bactericide, cancer-preventative, (herbicide, insecticide, insect repellant, pesticide), *irritant*, sedative, spasmolytic
linalool<br>leaf	antiseptic, antiviral and viricide, bactericide, cancer-preventative, fungicide, (insect repellant, perfumery, pesticide, termite repellant), sedative, spasmolytic, *tumor-promoter*
luteolin<br>shoot	aldose-reductase inhibitor, *antifeedant*, antihistaminic, antiinflammatory, antioxidant, antitussive, cancer-preventative, choleretic, diuretic, (pesticide), spasmolytic, xanthine oxidase inhibitor
menthol<br>leaf<br>1,000-24,385 ppm	*allergenic*, analgesic, anaesthetic, antibronchitic, antiinflammatory, antineuralgic, antiodontalgic, antipruritic, antirheumatic, antiseptic, antisinusitic, bradycardic, bronchomucolytic, bronchorrheic, *CNS depressant*, calcium-antagonist?, carminative, counterirritant, gastrosedative, myelorelaxant, (perfumery, pesticide), rubifacient, spasmolytic, vibriocide
menthone<br>plant<br>71-24,000 ppm	analgesic, antiseptic, cancer-preventative, sedative, spasmolytic
myrcene<br>leaf<br>10-2,485 ppm	analgesic, antimutanegic, antinociceptive, bactericide (insect repellant, insecticide, pesticide) spasmolytic
p--cymene<br>leaf, 9-29ppm	analgesic, antiflu, antiviral and virucide, antirheumatologic, bactericide, fungicide (herbicide, insect repellant, pesticide)
piperitone<br>leaf, 13-1285 ppm	antiasthmatic

pulegone<br>plant<br>100-24460 ppm	antiacetylcholinesterase inhibitor--anti Alzheimerian?, antihistaminic, antipyretic, cancer-preventative, *cerebrotoxic, hepatotoxic* (avifuge, herbicide, insecticide and insect repellant, pesticide), flea repellant, sedative
rosmarinic acid<br>plant 28,000 ppm<br>leaf 71-228 ppm	antianaphylactic, anticomplementary, antiedemic, *antigonadotrophic*, antihepatotoxic, antiherpetic, antiviral, viricide, antiinflamatory, antileukotrienic, antilipoperoxidant, antioxidant, antiradicular, antishock, antithreotropic, bactericide, cancer-preventative, (pesticide)
thujone<br>plant	*cerebrodepressant*, *convulsant, epileptigenic, hallucinogenic,* (herbicide, pesticide), *respirainhibitor*, spasmolytic
